# Distinct Roles of Plant Residues and Microbial Necromass in Soil Organic Carbon Accumulation and Stability in the *Alhagi sparsifolia* Community

**DOI:** 10.3390/plants15071030

**Published:** 2026-03-27

**Authors:** Mengfei Cong, Zhihao Zhang, Yang Hu, Akash Tariq, Jordi Sardans, Weiqi Wang, Xinping Dong, Guangxing Zhao, Jingming Yan, Josep Peñuelas, Fanjiang Zeng

**Affiliations:** 1 College of Ecology and Environment, Xinjiang University, Urumqi 830046, China; congmengfei95@163.com (M.C.); 15166470336@163.com (J.Y.); 2 Xinjiang Key Laboratory of Desert Plant Roots Ecology and Vegetation Restoration, Xinjiang Institute of Ecology and Geography, Chinese Academy of Sciences, Urumqi 830011, China; zhangzh@ms.xjb.ac.cn (Z.Z.); akash.malik786@mails.ucas.ac.cn (A.T.); dongxinping23@mails.ucas.ac.cn (X.D.); zhaoguangxing22@mails.ucas.ac.cn (G.Z.); 3 Key Laboratory of Ecological Safety and Sustainable Development in Arid Lands, Xinjiang Institute of Ecology and Geography, Chinese Academy of Sciences, Urumqi 830011, China; 4 Cele National Station of Observation and Research for Desert-Grassland Ecosystems, Cele 848300, China; 5 College of Resources and Environment, Xinjiang Agricultural University, Urumqi 830052, China; hy@xjau.edu.cn; 6 Consejo Superior de Investigaciones Cientificas (CSIC) Global Ecology Unit, CREAF-CSIC-UAB, Bellaterra, 08193 Barcelona, Catalonia, Spain; j.sardans@creaf.uab.cat (J.S.); josep.penuelas@uab.cat (J.P.); 7 Centre for Ecological Research and Forestry Applications (CREAF), Cerdanyola del Vallès, 08193 Barcelona, Catalonia, Spain; 8 University of Chinese Academy of Sciences, Beijing 100049, China; 9 Institute of Geography, Fujian Normal University, Fuzhou 350007, China; wangweiqi15@163.com

**Keywords:** *Alhagi sparsifolia*, soil organic carbon, plant residue, microbial necromass

## Abstract

In desert ecosystems, deep-rooted plants like *Alhagi sparsifolia* contribute not only to wind prevention and sand fixation but also to the transport of carbon into deep soil layers through their root systems. However, the sources and stabilization mechanisms of soil organic carbon (SOC) following plant carbon input remain unclear. This study investigated a dominant *A. sparsifolia* community at the southern edge of the Taklimakan Desert. We analyzed plant traits and the vertical distribution (0–200 cm) of SOC fractions—particulate organic carbon (POC), mineral-associated organic carbon (MAOC), and calcium/iron-bound organic carbon (Ca/Fe-OC)—along with carbon sources (microbial biomass, microbial necromass, and plant residue). As growth advanced, stem and root biomass increased, while leaf and thorn biomass remained stable. SOC and POC decreased by 5.38–29.43% with soil depth, whereas MAOC and Ca/Fe-OC increased by 32.34–48.15%. Plant residue contributed more to SOC (average 30.56%) than microbial necromass (8.28%), and both contributions increased by 9.60–167.68% with soil depth. No significant correlation was found between plant residue and SOC fractions, but a significant correlation with microbial necromass. In conclusion, although plant residues constitute the primary source of SOC in desert ecosystems, microbial necromassa exerts a stronger influence on SOC stability.

## 1. Introduction

In the global carbon cycle, plants in desert ecosystems play a significant role due to their extensive distribution. Although characterized by low primary productivity, the desert soil organic carbon (SOC) pool, owing to its long-term stability and sensitivity to climate change, has become a critical and vulnerable component in global carbon budget assessments [1]. However, the stability of SOC depends not only on its total amount but is also closely related to its specific chemical forms and physical protection mechanisms. Generally, SOC storage strongly relies on the retention of plant- and microbial-derived carbon [2]. Structural compounds in plant residues, such as lignin phenols, are considered important precursors for SOC formation due to their chemical recalcitrance and preferential accumulation in soils [3]. Conversely, soil microorganisms contribute directly to SOC formation through their turnover and metabolic products (primarily microbial necromass), which easily bind to minerals to create stable SOC for long-term storage [4]. Furthermore, based on physical protection mechanisms, chemical stability, and bioavailability, SOC can be further divided into particulate organic carbon (POC), mineral-associated organic carbon (MAOC), and more refined fractions such as calcium/iron-bound organic carbon (Ca/Fe-OC) [5]. Among these, POC primarily originates from partially decomposed plant residues, while MAOC and Ca/Fe-OC are regarded as more stable carbon pools dominated by microbial metabolites and formed through sorption onto reactive mineral surfaces [5]. Although significant progress has been made in understanding the composition, sources, and stabilization mechanisms of SOC, most studies have focused on humid climate regions. There remains a lack of in-depth understanding regarding the dynamic accumulation processes of SOC components in desert ecosystems, which hinders an accurate assessment of global carbon sequestration potential and persistence.

Desert environments are characterized by intense sunlight, extreme alternation between dryness and wetness, and sparse vegetation cover [1]. These conditions not only severely limit plant-derived carbon inputs but also profoundly alter the composition and function of soil microbial communities, potentially enhancing physicochemical protection mechanisms of SOC by promoting specific reactions of minerals such as calcium, iron, and clay minerals [6]. Typically, soil minerals can adsorb SOC molecules through electrostatic interactions, ligand exchange, ion bridging, and hydrogen bonding. Research on the Tibetan Plateau has shown strong correlations between SOC, microbial necromass, and mineral content, indicating that mineral-microbial interactions significantly influence SOC stability [7]. On the Loess Plateau, conservation tillage practices such as no-tillage enhances SOC sequestration through two mechanisms: by promoting the conversion of calcium carbonate to exchangeable calcium and associated calcium ion bridging, and by increasing mineral ions, organic-mineral complexes, and microbial biomass—thereby facilitating macroaggregate formation and improving the physical protection of SOC [8,9]. Additionally, fencing has been shown to increase SOC storage and strengthen its positive correlation with aggregate stability [10]. Drought can trigger the breakdown of macroaggregates and alter soil chemical properties, reducing SOC stability, while also modifying microbial community structure and activity, enhancing the transformation capacity of recalcitrant carbon [11]. Under arid conditions, limited root inputs and the interaction between carbon and mineral surfaces are confined to brief and shallow wet periods, leading to a disconnect between carbon concentration and its persistence [12]. Additionally, microbial necromass is widely recognized as important precursors of persistent organic matter in soil, particularly as key contributors to MAOC. Upon microbial death, the cell wall components in these residues not only exhibit high chemical stability but also show strong affinity for mineral surfaces, facilitating their association with soil minerals through physicochemical processes such as adsorption, thereby forming stable MAOC [4]. In both grassland and farmland ecosystems, studies have found a positive correlation between microbial necromass and MAOC [13]. However, existing studies have predominantly focused on specific regions, and the conclusions drawn may be influenced by local climate, parent material, and vegetation types, making them difficult to generalize to all desert ecosystems. A widely applicable model for SOC stability has yet to be established. Additionally, current research often emphasizes the role of single mechanisms, leaving a gap in understanding the interactive effects and relative contributions of plant residues, microbial necromass, and MAOC.

The desert-oasis ecotone is a typical ecological transition zone in arid landscapes, whose ecosystem structure and function play a crucial role in maintaining oasis stability and curbing desertification expansion [14]. In this ecologically fragile region, *A. sparsifolia* often exists as a dominant or constructive species, playing a vital ecological role in maintaining the stability of desert ecosystems and promoting the restoration of degraded land. As a typical deep-rooted desert plant, it exhibits strong drought and salinity tolerance, enabling it to adapt to extreme arid and infertile habitats. Its extensive root system effectively stabilizes sand, improves soil quality, and increases ground cover, making it indispensable in windbreak and sand fixation as well as soil and water conservation. Above ground, the plant is low-growing with well-developed and partially spine-like leaves to reduce transpiration; underground, it develops a deep root system reaching groundwater or moist soil layers, allowing efficient use of water resources. These characteristics not only ensure its stable survival in desert environments but also highlight its potential for application in vegetation restoration of degraded lands. By transporting atmospheric carbon into the soil (especially deep layers) via photosynthesis and regulating microbial processes, *A. sparsifolia* drives the conversion of carbon into stable forms, significantly influencing the stock, vertical distribution, and stability of SOC in the ecotone [15]. Therefore, this study focuses on the desert-oasis ecotone and aims to: (1) quantify the variations across soil depths and over time in different stability fractions of SOC, including POC, MAOC, and Ca/Fe-OC; (2) analyze the relative contributions of plant residues and microbial necromass to SOC using biomarker techniques; (3) investigate the relationships between SOC and different stability components. Based on the above understanding, we hypothesize that: (1) in desert ecosystems, SOC content and its components decrease with increasing soil depth; (2) soil microbial necromass are positively correlated with MAOC.

## 2. Results

### 2.1. Variations in Plant Characteristics Across Different Periods and Soil Layers

For the aboveground parts, stem biomass was the highest, ranging from 94 to 120 g plant^−1^, followed by leaves (75–79 g plant^−1^), while thorn biomass was the lowest (58–62 g plant^−1^). In terms of carbon content, leaves (43%) were significantly lower than stems and thorns (45%). As the growth period progressed, stem biomass gradually increased, showing a significant increase of 22–28% during the vigorous growth period and late growth period compared to the early growth period. However, leaf and thorn biomass did not change significantly throughout the entire growth period (Table 1).

Regarding root systems, root biomass in the 0–30 cm soil layer did not change significantly across different growth periods. In contrast, root biomass in the 30–60 cm and 60–100 cm soil layers increased significantly, increasing by 6.3–8.6% during the vigorous growth period and late growth period compared to the early growth period. Conversely, root biomass in the 100–200 cm soil layer decreased significantly, declining by 6.7–9.7% during the vigorous and late growth periods compared to the early stage. Overall, root carbon content in the 0–200 cm soil layer showed an increasing trend as the growth period advanced, with increases of 6.8–6.9% during the vigorous and late growth periods compared to the early stage (Table 1).

### 2.2. Variations in SOC and Its Fractions Across Soil Layers and Growth Periods

**Figure 1:**
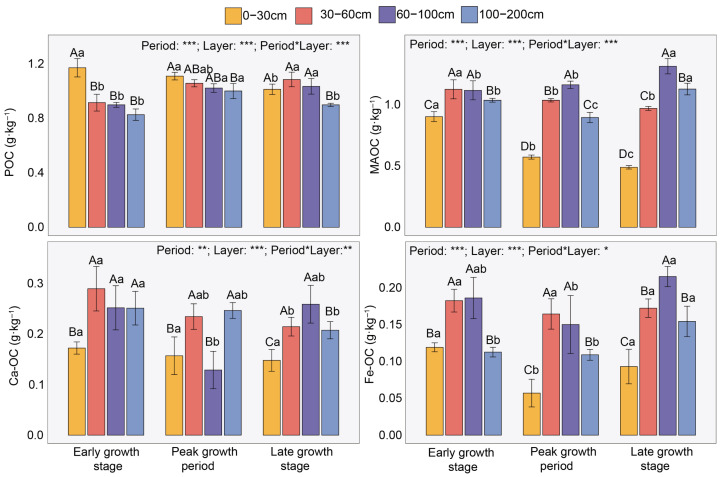
Variations in soil particulate organic carbon (POC), mineral-associated organic carbon (MAOC), calcium-bound organic carbon (Ca-OC), and iron-bound organic carbon (Fe-OC) across different periods and soil layers. Uppercase letters indicate significant differences among soil layers within the same period, while lowercase letters denote significant differences across periods within the same soil layer (*p* < 0.05). Based on two-way ANOVA, asterisks represent the following significance levels: * *p* < 0.05, ** *p* < 0.01, *** *p* < 0.001.

Overall, in the 0–200 cm soil layer, the contents of soil SOC and POC decreased gradually with increasing soil depth, whereas the MAOC content showed an increasing trend. Meanwhile, both POC and MAOC contents generally declined over time, particularly in the 0–30 cm layer (Figure 1). During the early growth stage, the POC content in the 30–200 cm layer decreased by 21.88–29.43% compared with that in the 0–30 cm layer. In contrast, during the peak and late growth stages, the POC content in the 100–200 cm layer was reduced by 5.38–9.85% relative to the 0–30 cm layer. Notably, across all observed periods, MAOC content in the 30–200 cm layer was 35.79–37.26% higher than that in the 0–30 cm layer (Figure 1). Specifically, at the early, peak, and late growth periods, MAOC content in the 30–200 cm layer was 14.80–24.70%, 56.42–103.05%, and 98.14–168.51% lower than that in the 0–30 cm layer, respectively (Figure 1).

Furthermore, Ca/Fe-OC contents in the 0–200 cm soil layer also showed a declining trend over time and generally increased with soil depth. Compared to the 0–30 cm layer, the contents of Ca-OC and Fe-OC in the 30–200 cm layer increased significantly by 35.42–32.34% and 48.15–28.39%, respectively (Figure 1). Among them, Ca-OC content (0.21 g·kg^−1^) was generally higher than Fe-OC content (0.14 g·kg^−1^) (Figure 1). Meanwhile, the contribution of Fe-OC and Ca-OC to SOC significantly increased with soil depth (Figure S1).

### 2.3. Variations in Soil Microbial PLFA Across Soil Layers and Growth Periods

**Figure 2:**
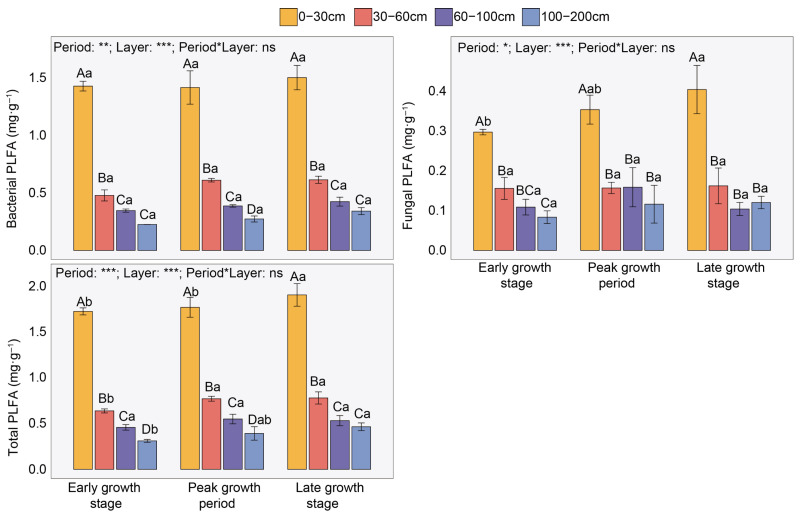
Variations in soil microbial phospholipid fatty acid (PLFA) across different periods and soil layers. Uppercase letters indicate significant differences among soil layers within the same period, while lowercase letters denote significant differences across periods within the same soil layer (*p* < 0.05). Based on two-way ANOVA, asterisks represent the following significance levels: ns *p* > 0.05, * *p* < 0.05, ** *p* < 0.01, *** *p* < 0.001.

Overall, soil bacterial PLFA content (mean 0.67 mg·g^−1^) was higher than that of fungi (0.19 mg·g^−1^). The contents of various PLFAs (including G^+^ bacteria, G^−^ bacteria, actinomycetes, arbuscular mycorrhizal fungi, and saprophytic fungi) decreased gradually with increasing soil depth in the 0–200 cm layer (Figure 2 and Figure S2). However, in the 0–30 cm layer, fungal and total microbial PLFA contents were significantly higher in the late growth period than in the early growth period, while bacterial PLFA showed no significant change over time. Specifically, compared with the 0–30 cm layer, the bacterial PLFA content in the 30–200 cm layer decreased by 60.86–80.69%, fungal PLFA content decreased by 59.72–78.54%, and total microbial PLFA content decreased by 55.01–69.71% (Figure 2).

### 2.4. Variations in Soil Microbial Necromass and Plant Residues Across Soil Layers and Growth Periods

**Figure 3:**
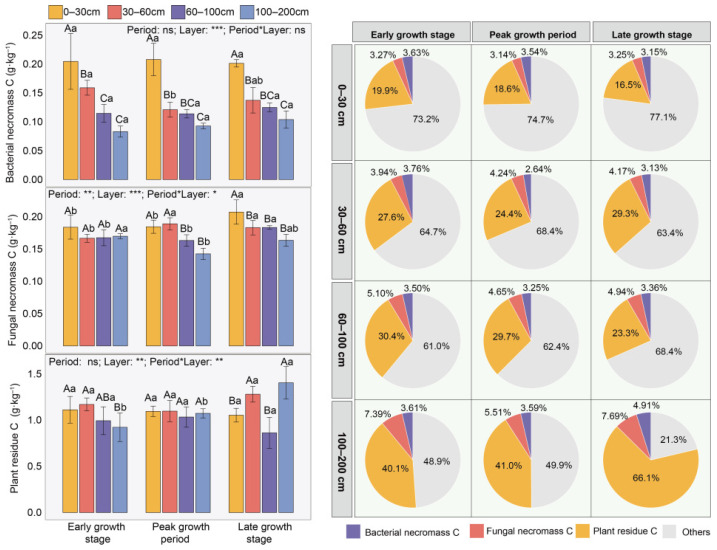
Variations in plant residue carbon and microbial necromass carbon across different periods and soil layers. Uppercase letters indicate significant differences among soil layers within the same period, while lowercase letters denote significant differences across periods within the same soil layer (*p* < 0.05). Based on two-way ANOVA, asterisks represent the following significance levels: ns *p* > 0.05, * *p* < 0.05, ** *p* < 0.01, *** *p* < 0.001.

The contents of lignin phenols and amino sugars showed no significant changes over time, but decreased with soil depth (Figure S3). At the early growth stage, plant residues in the 60–200 cm soil layer were significantly lower than those in the 0–60 cm layer; during the peak growth stage, no significant differences were observed among layers; in the late growth stage, plant residue carbon increased significantly in the 30–60 cm and 100–200 cm layers (Figure 3). Soil microbial necromass carbon likewise declined with increasing depth in the 0–200 cm soil layer. Compared with the 0–30 cm layer, bacterial necromass carbon in the 30–200 cm layer decreased by 31.99–54.38%, fungal necromass carbon decreased by 6.37–17.31%, and total microbial necromass carbon decreased by 19.59–36.43% (Figure 3 and Figure S4).

Overall, the contribution of plant residue carbon to SOC (30.57%) was higher than that of microbial necromass carbon (8.28%), with fungal necromass carbon (4.77%) slightly exceeding bacterial necromass carbon (3.51%) (Figure 3 and Figure S4). With increasing soil depth, the contributions of both plant residues and microbial necromass to SOC gradually increased, and the increase in plant residues (47.77–167.68%) was higher than that of microbial necromass (9.60–63.38%) (Figure 3).

### 2.5. Relationships Among SOC Fractions

**Figure 4:**
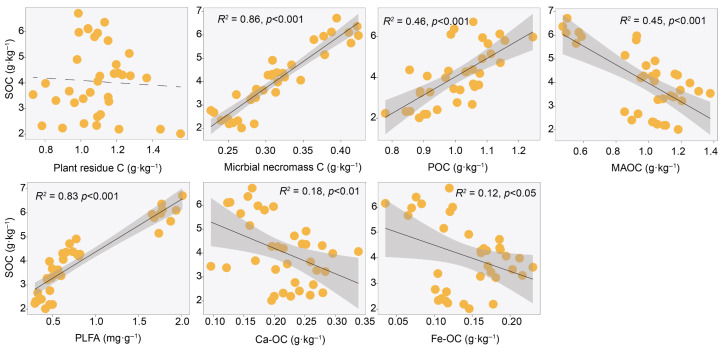
Relationships between soil organic carbon (SOC) and its components. The shaded area represents the 95% confidence interval, solid lines indicate significant regressions (*p* < 0.05), while dashed lines represent non-significant regressions (*p* > 0.05). Each dot represents an individual sample. POC: particulate organic carbon; MAOC: mineral-associated organic carbon; PLFA: phospholipid fatty acids; Ca-OC: calcium-bound organic carbon; Fe-OC: iron-bound organic carbon.

**Figure 5:**
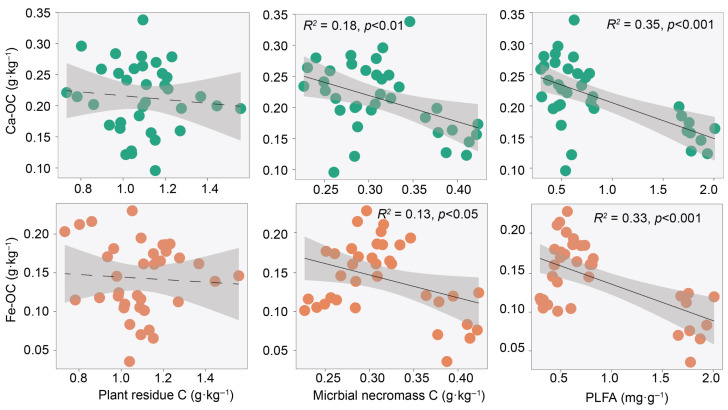
Relationships between particulate organic carbon (POC), mineral-associated organic carbon (MAOC), and plant residue carbon, microbial necromass carbon, phospholipid fatty acids (PLFA). The shaded area represents the 95% confidence interval, solid lines indicate significant regressions (*p* < 0.05), while dashed lines represent non-significant regressions (*p* > 0.05). Each dot represents an individual sample.

Overall, SOC showed no significant correlation with plant residues (*p* > 0.05), but was significantly positively correlated with microbial necromass, POC, and microbial PLFA (*R*^2^ = 0.46–0.86, *p* < 0.001), while being significantly negatively correlated with MAOC, Ca/Fe-OC (*R*^2^ = 0.12–0.45, *p* < 0.05) (Figure 4). Among these, Ca-OC explained more variation in SOC (*R*^2^ = 0.18) than Fe-OC (*R*^2^ = 0.12). Among SOC components, both POC and MAOC also exhibited no significant correlation with plant residues. POC was significantly positively correlated with microbial necromass and PLFA, and significantly negatively correlated with Ca-OC. In contrast, MAOC was significantly negatively correlated with microbial necromass and PLFA, but significantly positively correlated with Ca/Fe-OC (Figure S5). Plant residues showed no significant correlation with Ca/Fe-OC (*p* > 0.05), while microbial necromass and PLFA exhibited negative correlations with Ca/Fe-OC (*R*^2^ = 0.13–0.35, *p* < 0.05) (Figure 5). Additionally, plant root biomass was significantly positively correlated with MAOC and Ca-OC (*R*^2^ = 0.16–0.25, *p* < 0.05), but showed no significant correlations with other SOC fractions or sources (*p* > 0.05) (Figure S6).

## 3. Discussion

In the desert–oasis ecotone of the Taklamakan Desert, the biomass of *A. sparsifolia* stems was the highest among aboveground parts and increased progressively with advancing growth stages, whereas leaf and spine biomass showed no significant changes throughout the entire growth period (Table 1). This indicates that stems serve not only as supporting structures but also as primary long-term storage organs for photosynthates. As the growth period transitions from vegetative to reproductive growth, the plant tends to allocate more photosynthates to longitudinal growth and lignified thickening of stems to occupy a higher light-resource niche, which aligns with theoretical models of plant biomass allocation [16]. Carbon content in leaves was significantly lower than in stems and thorns, primarily due to differences in physiological function and chemical composition: leaves are rich in proteins and soluble metabolites, whereas lignified stems and cutinized spines contain more cellulose, hemicellulose, and lignin—the latter typically having higher carbon concentrations [17]. Root biomass in the 30–100 cm soil layer increased significantly as the growth period advanced (Table 1), reflecting the plant’s enhanced demand for stable water sources and deep nutrients during vigorous growth, which drives the extension of fine roots into deeper layers—a phenomenon particularly common in arid and semi-arid ecosystems. However, in the 100–200 cm deep soil layer, root biomass decreased markedly, with some roots potentially senescing and dying by the end of the growth period, leading to a temporary reduction in biomass [18]. In summary, *A. sparsifolia* concentrates resources on stem growth aboveground to support canopy expansion, while belowground it enhances deep root development to continuously explore water sources in deeper soil layers, reflecting an important survival strategy for plants adapting to seasonal arid environments.

Consistent with our first hypothesis, both SOC and POC contents in the desert ecosystem decreased significantly with increasing soil depth along the 0–200 cm soil profile (Figure 1). Typically, the surface soil is the main input zone for plant litter, root exudates, and dead roots [19]. These fresh organic materials form POC after initial decomposition. POC consists of physically large, weakly mineral-associated carbon with high lability, which are readily utilized by microorganisms [5]. Therefore, POC is primarily concentrated in the topsoil and declines rapidly with depth. Moreover, in arid regions, plant root biomass usually decreases sharply with depth. The carbon input to deeper soil layers mainly relies on limited deep root exudates and the downward leaching of dissolved SOC from the surface, yet its flux is much lower than that in the topsoil [19]. Similar findings have been observed in studies conducted in both arid and humid regions [20,21]. However, contrary to the first hypothesis, we found that MAOC and Fe/Ca-OC content both increased significantly with soil depth in the 0–200 cm layer (Figure 1). Although this result differs from previous studies in other ecosystems [20], research in desert grasslands has also shown that MAOC content in the 20–40 cm soil layer is higher than that of POC [22]. Additionally, studies in the Inner Mongolia grassland indicate that the proportion of MAOC in SOC increases with depth in the 0–50 cm soil layer [23]. As soil depth increases, soil texture usually becomes more clayey (i.e., clay content rises), and the contents of minerals such as Fe-oxides and calcium carbonate (CaCO_3_) also increase. These minerals possess large specific surface areas and abundant reactive sites, enabling them to bind with soluble organic carbon through adsorption, complexation, or co-precipitation, thereby forming MAOC or more stable Fe/Ca-OC [24]. Such binding greatly slows down microbial decomposition of SOC. Furthermore, in desert ecosystems, especially in sandy soils, dissolved organic carbon derived from surface plant litter or root exudates can migrate downward with water during occasional precipitation events. During this movement, the labile fraction is consumed by microorganisms in the upper layers, while the remaining recalcitrant fraction is intercepted and stabilized upon encountering deeper clay and Fe/Ca minerals, thus promoting the accumulation of MAOC in deeper soil layers.

Regarding the sources of SOC, we found that both plant residues and microbial necromass carbon decreased with soil depth in the 0–200 cm soil layer (Figure 3), which is consistent with our first hypothesis. This vertical pattern aligns with observations from other ecosystems (alpine meadow, typical steppe, desert steppe, etc.) [23,25]. First, surface litter inputs and root activities in topsoil provide the most abundant carbon sources for shallow layers. With increasing depth, live root biomass and root exudates generally decline exponentially, leading to substantially lower carbon inputs into deeper soil layers compared to the surface [19]. Therefore, the reduction in plant residue carbon is primarily controlled by the vertical gradient of carbon input. Second, topsoil usually exhibits more favorable temperature, moisture, and oxygen conditions, along with more abundant labile organic substrates, thereby sustaining higher microbial activity and turnover rates. Although microbial necromass itself is an important contributor to the stable carbon pool, its production and accumulation still depend on active microbial processes. In deeper soil layers, environmental stresses such as low temperature, oxygen limitation, and scarcity of nutrients and energy substrates strongly suppress microbial activity and growth, resulting in decreased microbial biomass carbon and consequently lower microbial necromass carbon production. Our results also confirm that microbial biomass (PLFA) decreases significantly with soil depth (Figure 2). However, in the 0–200 cm soil layer, the contributions of both plant residues and microbial necromass to SOC increased with depth (Figure 3), which is consistent with the findings of studies on grassland and forest soils at the global scale [26]. First, diverse SOC sources in surface soil (e.g., microbial extracellular polymers, etc.) may dilute the relative contribution proportions of plant residue and microbial necromass [27]. Moreover, microorganisms are a major source of nitrogen; under nitrogen-limited conditions, they may preferentially degrade or reuse microbial necromass, potentially reducing its preservation and lowering microbial conversion efficiency [28]. In contrast, in deeper soil layers, although direct plant residue inputs are reduced, fine root turnover and rhizodeposition processes (e.g., root exudates) continue to supply organic carbon. These energy-rich compounds can stimulate microbial activity, whereby microbial necromass becomes effectively stabilized while microbes assimilate rhizosphere carbon sources [4]. Additionally, with increasing depth, reduced temperature fluctuations and nutrient poverty limit microbial activity. Such conditions inhibit the decomposition of both fresh plant residues and microbial necromass. Furthermore, microbial necromass exhibits higher biochemical stability and greater resistance to further degradation, allowing it to accumulate on mineral surfaces and become a key component of the deep SOC pool [4].

In arid desert ecosystems, plant residue carbon in the SOC pool is significantly higher than microbial necromass carbon (Figure 3). Although the result that plant residue carbon content is higher than microbial necromass carbon content differs from previous findings in typical grasslands [29], the dominance of plant residue carbon in desert ecosystems is primarily due to the severe suppression of microbial decomposition processes under extreme aridity. Low water content significantly reduces microbial activity and turnover rates, leading to extremely slow decomposition of fresh organic matter such as litter and plant roots. This process results in a relatively low efficiency of microbial necromass formation in deserts, and the contribution of microorganisms to the stable SOC pool is therefore lower than in humid ecosystems [30]. Secondly, previous studies have shown that the roots of *A. sparsifolia* can grow to a depth of over 2.5 m within a year [31]. To seek water in barren sandy soil, the roots grow continuously, and after death, their residues remain directly in the soil. Additionally, under harsh environmental conditions, the roots of *A. sparsifolia* have a high lignin content, making them more resistant to decomposition under drought stress and allowing them to persist in the soil as residues for extended periods [32]. Meanwhile, the fungal-dominated pattern within microbial necromass carbon (Figure 3) reflects the stronger physiological and ecological adaptability of fungi compared to bacteria under drought stress. This is consistent with previous studies: in global farmland, grassland, and forest ecosystems, fungal necromass account for more than 65% of the total microbial necromass, significantly exceeding bacterial necromass [26]. On the one hand, unlike bacteria, fungi can utilize recalcitrant compounds and high-carbon resources to synthesize their own biomass, giving them a greater competitive advantage in nutrient acquisition [13]. Fungi, especially saprophytic soil fungi, possess extensive hyphal networks that can span heterogeneous dry and wet microsites in the soil, accessing spatially scattered water and nutrients, thereby maintaining certain metabolic activity [33]. On the other hand, the main component of fungal cell walls, chitin, exhibits higher chemical stability than the peptidoglycan of bacterial cell walls, making fungal necromass more resistant to further decomposition in soil and thus resulting in longer residence times and higher accumulation [34]. Moreover, in barren desert soils, fungi generally have higher carbon use efficiency, meaning they convert more absorbed carbon into their own biomass rather than respiratory losses, which increases the potential input of fungal necromass at the source.

We found that POC was significantly positively correlated with SOC, whereas MAOC showed a significant negative correlation with SOC (Figure 4). In desert ecosystems, sparse vegetation and limited litter input result in slow decomposition. Plant residues primarily form POC after physical fragmentation. As a relatively active carbon pool, the content of POC directly reflects the amount of recent plant carbon inputs [35]. In extremely arid desert environments, moisture is the key limiting factor for microbial activity. Low moisture conditions strongly suppress microbial decomposition and transformation processes, leading to very low efficiency in converting POC to MAOC [4]. Therefore, changes in total SOC are largely driven by the input and accumulation of POC, which accounts for the positive correlation between them; meanwhile, MAOC and Fe/Ca-OC accordingly exhibit a negative correlation with SOC (Figure 4).

Fitting analysis found no significant correlation between plant residue and SOC, whereas microbial necromass showed a significant positive correlation with SOC (Figure 4). Global-scale studies have similarly confirmed that microbial necromass are more critical than plant residues for SOC accumulation and exhibit a stronger correlation with SOC [36]. This indicates that the input of fresh plant residues is not a direct factor determining the long-term storage of SOC; instead, microbial necromass and their transformation processes contribute more critically to SOC accumulation [27]. First, although plant residues account for a relatively high proportion of SOC (Figure 3), their components are primarily easily decomposed by microorganisms, resulting in the release of most carbon as CO_2_; only a small component of recalcitrant components or products transformed by microorganisms can enter relatively stable carbon pools [27]. Second, microbial necromass are considered an important direct source of stable SOC. Microorganisms convert assimilated simple carbon sources into their own biomass through anabolism. The microbial necromass formed after their death, due to the inherent stability of their chemical structures and strong affinity with soil minerals, are more readily preserved in soil over the long term [4,24]. Plant residue carbon was not significantly correlated with Fe/Ca-OC, while microbial necromass carbon was closely related to the latter (Figure 5), further confirming the close association between microbial necromass and soil minerals. However, contrary to our second hypothesis, we found that microbial necromass were negatively correlated with MAOC and Fe/Ca-OC (Figure 5). This result differs from previous research, which reported a positive correlation between microbial necromass and MAOC in grassland ecosystems [37]. This difference may reflect the unique water limitations in desert ecosystems and their regulatory role in SOC turnover. First, the extremely arid environment directly constrains microbial transformation efficiency. Microbial activity in desert soils is strongly inhibited by long-term water availability limitations [38]. The limited water availability not only hinders the diffusion of microbial secretions and enzymatic reactions but also shifts microbial metabolism toward maintenance rather than growth and reproduction, thereby severely suppressing the production efficiency of microbial necromass carbon [39]. Under such low turnover rates, the amount of microbial necromass produced is too low to generate sufficient precursor materials for binding to mineral surfaces. Second, the migration pathway of microbial necromass to mineral fractions is impeded. In desert ecosystems, due to the lack of water as a medium, microbial necromass carbon may remain largely in unprotected particulate organic fractions. These necromass, under resource-limited conditions, instead become substrates for microbial decomposition and are repeatedly mineralized, preventing effective adsorption and stabilization on mineral surfaces and ultimately failing to enter the MAOC pool [5]. Third, desert soils are often rich in minerals such as calcium carbonate and free iron oxides, but the adsorption sites on these minerals may already be occupied by other carbon sources (e.g., dissolved organic matter). Taken together, in desert ecosystems, water limitation not only reduces the production efficiency of microbial necromass but also weakens the pathway for its migration and transformation into mineral fractions, resulting in microbial necromass being primarily retained in the more active particulate fractions.

## 4. Materials and Methods

### 4.1. Study Area Overview

The study area is located at the junction between the Cele Oasis and the southern margin of the Taklimakan Desert in China (37°00′57″ N, 80°43′45″ E, altitude 1353 m). The climate is extremely arid, characterized by an annual average temperature of 12.7 °C, an annual average precipitation of only 35 mm, and a potential evaporation reaching 2600 mm. The groundwater table is 13–15 m deep. According to the Genetic Soil Classification of China, the soil type is classified as aeolian sandy soil, corresponding to Arenosols in the World Reference Base for Soil Resources (WRB) classification [40]. The area is widely covered with *A. sparsifolia* as the dominant plant species, whose root system can extend up to 20 m deep to adapt to the harsh desert environment [15].

### 4.2. Experimental Setup and Sample Collection

Sampling was conducted during three key plant growth stages in 2022: early growth stage (14 June), peak growth stage (11 July), and late growth stage (18 September). In areas with extensive distribution of *A. sparsifolia* within the study region, three 100 m × 100 m plots were randomly established, serving as three independent replicate plots. Soil samples were collected from depths of 0–30 cm, 30–60 cm, 60–100 cm, and 100–200 cm. Specifically, five 1 m × 1 m quadrats were randomly selected in each plot, and soil from the same depth in the five quadrats was mixed into one composite sample to represent the soil characteristics at that depth in the plot. Thus, one composite sample per depth per plot was obtained at each sampling stage, with three plots providing three replicates. A total of 36 composite soil samples were collected (3 growth stages × 4 soil layers × 3 replicate plots). The samples were divided into two portions based on research objectives: one portion was freeze-dried for phospholipid fatty acid (PLFA) analysis, and the other was air-dried for physicochemical property determination.

### 4.3. Determination of Plant and Soil Properties

After collection, plant samples were separated by organ (roots, stems, leaves, thorns), placed in paper sample bags, dried in a forced-air oven at 105 °C to constant weight, and weighed to record biomass. Carbon content in plant samples was determined using an elemental analyzer (Elementar vario MACRO cube, Langenselbold, Germany) [41].

Soil moisture (SM) was determined by the oven-drying method. Soil pH and electrical conductivity (EC) were measured using a multiparameter electrode (Mettler Toledo FE28, Greifensee, Switzerland). Soil organic carbon (SOC) was determined by the external-heat potassium dichromate (K_2_Cr_2_O_7_) oxidation method [42]. Soil inorganic nitrogen (NH_4_^+^ and NO_3_^−^) was analyzed with a continuous flow analysis system (SEAL AA3, Norderstedt, Germany); total nitrogen (TN) was quantified using a Kjeldahl nitrogen analyzer (FOSS Kjeltec 8400, Hillerød, Denmark) [43]. Available phosphorus (P) was extracted with sodium bicarbonate (NaHCO_3_) solution, while total phosphorus was extracted by sodium hydroxide (NaOH) fusion, and both were quantified with a spectrophotometer (Shimadzu UV-2450, Kyoto, Japan) [44]. Available potassium (K) was extracted using ammonium acetate (CH_3_COONH_4_) solution; for total potassium determination, samples were digested with a mixture of nitric acid (HNO_3_), perchloric acid (HClO_4_), and hydrofluoric acid (HF) to prepare the test solution, and then measured by flame photometry (Shanghai Youke FP6400, Shanghai, China).

### 4.4. Determination of POC and MAOC

The physical fractions of SOC were determined using the wet-sieving method. Undisturbed soil samples were air-dried naturally, after which gravel and plant residues were removed. A 20 g soil sample was mixed with a dispersant (sodium hexametaphosphate solution) and fully dispersed on a shaker. The entire suspension was poured onto a 53 μm standard sieve and rinsed thoroughly with distilled water while continuously shaking until no soil particles passed through. The materials retained on and passed through the sieve were separately transferred into crucibles, dried, and weighed. The fraction retained on the sieve represented POC, while the fraction passing through represented MAOC. The SOC content in each fraction was then measured and multiplied by the respective mass proportion of POC or MAOC to calculate their actual contents [45].

### 4.5. Determination of Fe/Ca-OC

A 2 g soil sample was weighed and mixed with 20 mL of 0.5 M neutral Na_2_SO_4_ solution. After oscillation, standing, and centrifugation, the residue was collected. It was then repeatedly washed with diluted Na_2_SO_4_ solution (1%) and deionized water until the washing solution became colorless. The organic carbon content of the residue was measured, and the carbon released during the Na_2_SO_4_ extraction was regarded as Ca-OC [46].

Fe-OC was determined by reduction extraction using the dithionite-citrate-bicarbonate (DCB) method: 0.25 g of soil sieved through 2 mm was placed into a 50 mL centrifuge tube with 15 mL of a mixed solution of sodium citrate (Na_3_C_6_H_5_O_7_·2H_2_O) and sodium bicarbonate (NaHCO_3_), and incubated in an 80 °C water bath. Then, 0.25 g of sodium dithionite (Na_2_S_2_O_4_) was added, and the mixture was maintained at 80 °C for 15 min. After cooling to room temperature, it was centrifuged at 3000 r/min for 30 min, and the supernatant was removed; this extraction procedure was repeated three times. The residue was subsequently washed and centrifuged three times with 15 mL of deionized water. Finally, the residue was dried, ground, and its organic carbon content was determined, which represented the iron-bound organic carbon content [47].

### 4.6. Determination of Microbial Biomass

After freeze-drying, approximately 6 g of soil sample was accurately weighed and transferred into a correspondingly labeled 80 mL capped glass centrifuge tube. Subsequently, 18 mL of a chloroform-methanol-phosphate buffer mixture (1:2:0.8, *v*/*v*/*v*) and internal standard 1 were added, followed by shaking at 160 rpm in the dark for 2 h. Then, 5 mL of chloroform was added to a separatory funnel, and after centrifugation (2000 rpm, 15 min), the supernatant was collected. The soil residue was extracted once more, and the supernatants were combined. The combined solution was shaken in a separatory funnel for 15 min, and the lower layer was collected into a 50 mL round-bottom flask. An additional 6 mL of chloroform was added, and the mixture was shaken and left to separate overnight in the dark. The lower phase was collected and purified through a silica gel column, eluting successively with chloroform, acetone, and methanol. The eluate was concentrated to approximately 0.5 mL by rotary evaporation at 40 °C, transferred to a derivatization vial, rinsed with methanol, and dried under a nitrogen stream. Alkaline methanol hydrolysis (0.5 M NaOH, 100 °C) and BF3-methanol derivatization (80 °C) were then performed. After neutralization, the mixture was extracted with n-hexane, and the extracts were combined and evaporated to dryness under nitrogen. Finally, internal standard 2 and toluene were added to dissolve the residue, and the microbial PLFA content was determined using gas chromatography (Agilent 6890A, Santa Clara, CA, USA) [48].

### 4.7. Determination of Lignin Phenols

Lignin phenols were selected as biomarkers for plant residue carbon and were determined using a modified alkaline CuO oxidation method. The procedure was as follows: First, approximately 0.5–1.0 g of freeze-dried and ground soil sample was weighed into a polytetrafluoroethylene-lined stainless steel reaction vessel. Then, 1.0 g CuO, 0.1 g ferrous ammonium sulfate (Fe(NH_4_)_2_(SO_4_)_2_), 50 μL of internal standard, and 15 mL of 2 M NaOH solution were added. After purging with nitrogen to remove air, the vessel was sealed and heated in an oven at 170 °C for 2 h. After the reaction, the mixture was processed through centrifugation, extraction, nitrogen blowing, and derivatization. Finally, the lignin phenols were qualitatively and quantitatively analyzed using a gas chromatograph (Agilent 6890A, Santa Clara, CA, USA). The target analytes included vanillyl (V: vanillin, acetovanillone, vanillic acid), syringyl (S: syringaldehyde, acetosyringone, syringic acid), and cinnamyl (C: ferulic acid, p-coumaric acid) phenolic monomers. Plant-residue carbon was calculated based on the contents of lignin phenol monomers, namely vanillyl (V), syringyl (S), and cinnamyl (C) types [2]. Assuming that the release efficiency of C-type lignin phenols reaches 100% during oxidation, plant residue carbon was quantified using the following formula:

(1) $$\mathrm{Plant}\;\mathrm{residue}\;\mathrm{carbon}\;=\;\frac{\frac{V}{33.3\%}\;+\;\frac{S}{\;90\%}\;+\;C}{0.03\times \mathrm{SOC}}\times 100\%$$

where V, S, and C represent the carbon contents of vanillyl (V), syringyl (S), and cinnamyl (C)-type lignin phenols, respectively; SOC denotes the soil organic carbon content. The values 33.3% and 90% correspond to the estimated release efficiencies of V-type and S-type phenols in alkaline CuO oxidation, respectively. The coefficient 0.03 represents the minimum lignin content factor in major plant residues.

### 4.8. Determination of Amino Sugars

Amino sugars were selected as biomarkers of microbial necromass carbon and extracted from soil using an acid hydrolysis method, primarily including glucosamine (GluN), galactosamine (GalN), and muramic acid (MurA). The procedure was as follows: 0.3–0.5 g of soil sample was weighed and hydrolyzed with 10 mL of 6 M HCl at 105 °C for 8 h. After cooling, 100 μL of 1 μg/μL myo-inositol was added as an internal standard. The mixture was filtered, and the filtrate was evaporated to dryness using a rotary evaporator. Subsequently, after centrifugation, derivatization, and nitrogen blowing, the extracts were analyzed by gas chromatography (Agilent 6890A, Santa Clara, CA, USA). Bacterial necromass carbon and fungal necromass carbon were calculated using the following formulas:

(2) Bacterial necromass carbon = MurA×45

(3) $$\mathrm{Fungal}\;\mathrm{necromass}\;\mathrm{carbon}=(\frac{\mathrm{GluN}}{179.17}-2\times \frac{\mathrm{MurA}}{251.23})\times 179.17\times 9$$

where 179.17 and 251.23 represent the molecular weights (g·mol^−1^) of GluN and MurA, respectively; the coefficients 9 and 45 are conversion factors for fungal and bacterial dead biomass carbon, respectively. Total microbial necromass carbon was estimated as the sum of fungal and bacterial dead biomass carbon [2].

### 4.9. Data Analysis

All data analyses were performed using the R (version 4.5.1). Prior to analysis, the Shapiro-Wilk test and Levene’s test from the “car” package (version 3.1.3) were used to assess normality and homogeneity of variance, respectively, and all data met the assumptions. Subsequently, two-way ANOVA was performed using the “tidyverse” package (version 2.0.0) to examine the effects of soil layer, period, and their interaction. For variables with significant differences, one-way ANOVA followed by Tukey’s post-hoc test (*p* < 0.05) was used to compare soil properties. In regression analysis, to control the false positive rate in multiple testing, *p*-values were adjusted using the false discovery rate (FDR) method from the “fdrtool” package (version 1.2.18), as the FDR approach can better balance statistical power when controlling errors.

## 5. Conclusions

This study systematically analyzed SOC components in the oasis-desert ecotone at the southern margin of the Taklimakan Desert, revealing the vertical differentiation characteristics and stabilization mechanisms of SOC in arid zones. With increasing soil depth, the more labile POC and microbial biomass decreased significantly, while MAOC and Fe/Ca-OC contents increased markedly. This suggests that the turnover of SOC in surface soils is more influenced by biological processes. In contrast, the long-term stability of SOC in the deeper layers of soil primarily relies on physicochemical associations with minerals, particularly the protective effects of calcium and iron oxides. Notably, plant residue carbon contributed more than microbial necromass carbon consistently to the SOC pool throughout the profile, but plant residue carbon showed no significant correlation with SOC or its components. In contrast, microbial necromass was positively correlated with POC, while negatively correlated with MAOC and Fe/Ca-OC. These findings demonstrate that mineral protection, rather than microbial transformation, is the dominant mechanism for deep-layer MAOC accumulation. This insight provides key mechanistic parameters for carbon cycling models in arid regions. It also offers a reference for ecological restoration in global arid regions, prioritizing practices that maintain or enhance mineral-associated carbon pools when developing vegetation restoration strategies, thereby maximizing long-term soil carbon sequestration potential.

## Figures and Tables

**Table 1:** Variations in plant characteristics across different periods and soil layers.

Period	Plant Organs	Soil Layer	Root Biomass (g Plant^−1^)	C (%)
Early growth stage	Root	0–30 cm	94.49 ± 3.71 Ba	44.24 ± 0.66 Ab
		30–60 cm	89.14 ± 3.59 Bb	43.68 ± 0.72 Ab
		60–100 cm	79.97 ± 5.24 Cb	43.83 ± 0.19 Ab
		100–200 cm	103.39 ± 3.93 Aa	43.30 ± 1.46 Ab
	Stem		94.12 ± 3.37 Ac	44.58 ± 0.29 Aa
	Thorn		58.00 ± 1.00 Ca	44.74 ± 0.15 Aa
	Leaf		77.33 ± 2.52 Ba	43.12 ± 0.55 Ba
Peak growth stage	Root	0–30 cm	98.49 ± 7.58 Aa	46.4 ± 0.65 ABa
		30–60 cm	95.81 ± 4.97 Aa	46.50 ± 2.30 Aa
		60–100 cm	86.64 ± 3.97 Ba	45.69 ± 0.79 ABa
		100–200 cm	96.72 ± 1.90 Ab	45.03 ± 0.97 Ba
	Stem		114.63 ± 4.75 Ab	45.33 ± 0.11 Aa
	Thorn		58.67 ± 3.51 Ca	45.25 ± 0.07 Aa
	Leaf		75.00 ± 5.00 Ba	42.89 ± 0.21 Ba
Late growth stage	Root	0–30 cm	93.83 ± 2.82 ABa	45.8 ± 0.78 Aa
		30–60 cm	94.81 ± 8.96 Aa	45.27 ± 0.51 Aa
		60–100 cm	85.30 ± 1.66 Ba	45.74 ± 0.78 Aa
		100–200 cm	93.39 ± 6.64 ABb	45.97 ± 0.29 Aa
	Stem		120.03 ± 1.05 Aa	45.93 ± 0.31 Aa
	Thorn		62.00 ± 1.00 Ca	45.12 ± 0.42 Ba
	Leaf		78.67 ± 2.08 Ba	42.62 ± 0.03 Ca

Uppercase letters indicate statistically significant differences (*p* < 0.05) among different organs/soil layers within the same sampling period, while lowercase letters indicate significant differences (*p* < 0.05) across sampling periods within the same organ/soil layer.

## Data Availability

The original contributions presented in this study are included in the article. Further inquiries can be directed to the corresponding author.

## Supplementary Materials

The following supporting information can be downloaded at: https://www.mdpi.com/article/10.3390/plants15071030/s1, Figure S1: Variations in the contributions of soil calcium-bound organic carbon (Ca-OC) and iron-bound organic carbon (Fe-OC) to soil organic carbon (SOC) across different periods and soil layers; Figure S2. Variations in soil microbial phospholipid fatty acid (PLFA) across different periods and soil layers; Figure S3. Variations in soil amino sugars and lignin phenols across different periods and soil layers; Figure S4. Variations in soil microbial necromass carbon across different periods and soil layers; Figure S5. Relationships between particulate organic carbon (POC), mineral-associated organic carbon (MAOC) and plant residue carbon, microbial necromass carbon, phospholipid fatty acids (PLFA), calcium-bound organic carbon (Ca-OC), iron-bound organic carbon (Fe-OC); Figure S6. Relationships between plant root biomass and particulate organic carbon (POC), mineral-associated organic carbon (MAOC) and plant residue carbon, microbial necromass carbon, phospholipid fatty acids (PLFA), calcium-bound organic carbon (Ca-OC), iron-bound organic carbon (Fe-OC); Table S1: Dynamics of soil characteristics.
