# Supplementary material for: Distinct Roles of Plant Residues and Microbial Necromass in Soil Organic Carbon Accumulation and Stability in the Alhagi sparsifolia Community
Source: Plants (Basel). 2026 Mar 27;15(7):1030. doi: 10.3390/plants15071030 (PMC13075010; doi:10.3390/plants15071030)
Supplement: Supplementary file 1 [file plants-15-01030-s001.zip › plants-4212993-supplementary.pdf]

Supplementary Information

Table S1. Dynamics of soil characteristics

Periods	Soil layer	Miosture (%)	pH	EC (μs/cm)	SOC (g/kg)	NH_4^+ (mg/kg)
Early growth stage	0-30cm	0.17±0.03Da	8.41±0.02Ab	419.67±7.64Bc	5.64±0.43Abc	1.21±0.14Bb
	30-60cm	0.37±0.07Cab	8.6±0.03Ab	779±14.11Aab	4.25±0.16Ba	2.26±0.09Aa
	60-100cm	0.58±0.03Bb	8.43±0.06Ab	347.33±95.71Cb	3.27±0.04Cb	2.73±0.08Acd
	100-200cm	0.7±0.07Ab	8.84±0.05Ab	120.73±9.41Db	2.3±0.05Dab	1.11±0.08Bb
Peak growth stage	0-30cm	0.2±0.04Da	8.27±0.05Ab	602±21.52Ba	5.91±0.24Ab	1.63±0.22Cab
	30-60cm	0.4±0.05Ca	8.54±0.12Ab	740.33±16.04Ab	4.52±0.34Ba	2.54±0.54Ba
	60-100cm	0.66±0.03Ba	8.38±0.05Ab	423.33±19.14Ca	3.5±0.16Cab	4.59±0.23Ab
	100-200cm	0.8±0.02Aa	8.95±0.02Ab	107.03±5.58Db	2.62±0.18Da	1.49±0.15Cb
Late growth stage	0-30cm	0.21±0.01Da	8.48±0.03Ab	556.67±32.72Bb	6.4±0.31Aa	0.33±0.19Cc
	30-60cm	0.39±0.04Ca	8.54±0.05Ab	818±24.33Aa	4.4±0.28Ba	1.51±0.17Bb
	60-100cm	0.58±0.04Bb	8.66±0.03Ab	337.67±5.69Cb	3.72±0.23Ca	2.24±0.15Ad
	100-200cm	0.75±0.05Aab	9.03±0.07Ab	114.27±4.33Db	2.13±0.1Db	0.3±0.06Cc

Periods	NO_3^- (mg/kg)	Available P (mg/kg)	Available K (mg/kg)	Total N (g/kg)	Total P (g/kg)	Total K (g/kg)
Early growth stage	12.26±0.51Bc	2.23±0.26Ad	293±1.73Ab	0.16±0.01Aa	0.58±0.02Ab	18.42±0.22Abc
	22.21±0.99Ac	1.23±0.1Cb	140±7Bc	0.17±0.01Aa	0.58±0Abc	18.58±0.22Aa
	6.8±0.4Cc	1.44±0.28Ca	112.33±1.53Cc	0.16±0.01ABab	0.58±0.01Ab	18.25±0.26Ac
	2.29±0.16Da	1.83±0.17Ba	113.67±2.31Ca	0.13±0Ba	0.57±0.01Ad	18.26±0.09Ac
Peak growth stage	28.75±2.11Bab	6.19±0.67Aa	278±7.21Ac	0.13±0.01Bb	0.62±0Aa	18.54±0.17Aab
	36.27±6.23Ab	0.88±0.04Cb	198.33±2.52Ba	0.18±0.01Aa	0.58±0.01Bc	18.58±0.25Aa
	18.16±0.21Ca	1.14±0.25BCab	124.33±0.58Cb	0.18±0.02Aa	0.61±0Aa	18.71±0.14Ab
	5.2±0.2Da	1.41±0.07Bb	104.33±0.58Db	0.13±0.01Ba	0.62±0.01Aa	18.4±0.42Ac
Late growth stage	31.72±2.14Ba	3.84±0.07Ab	280.33±2.31Ac	0.14±0Bb	0.62±0.02Aa	18.12±0.16Ac
	41.75±3.03Aa	1.7±0.21Ba	195.33±9.29Ba	0.17±0.01Aa	0.6±0.01Bab	18.33±0.29Aa
	15.91±2.2Cab	0.78±0.04Cc	104±4Cd	0.16±0.04ABab	0.62±0.01ABa	18.23±0.24Ac
	4.42±0.89Da	0.73±0.18Cc	80.67±1.53Dc	0.15±0.01Ba	0.6±0.01Bbc	18.17±0.2Ac

Uppercase letters indicate statistically significant differences ($p < 0.05$) among different soil layers within the same sampling period, while lowercase letters indicate significant differences ($p < 0.05$) across sampling periods within the same soil layer.

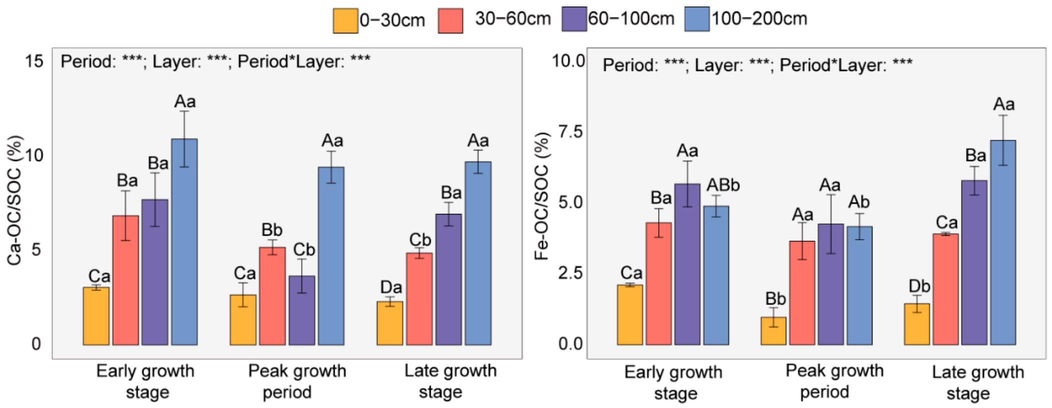

Figure S1. Variations in the contributions of soil calcium-bound organic carbon (Ca-OC) and iron-bound organic carbon (Fe-OC) to soil organic carbon (SOC) across different periods and soil layers. Uppercase letters indicate significant differences among soil layers within the same period, while lowercase letters denote significant differences across periods within the same soil layer ($p < 0.05$). Based on two-way ANOVA, asterisks represent the following significance levels: * $p < 0.05$, ** $p < 0.01$, *** $p < 0.001$.

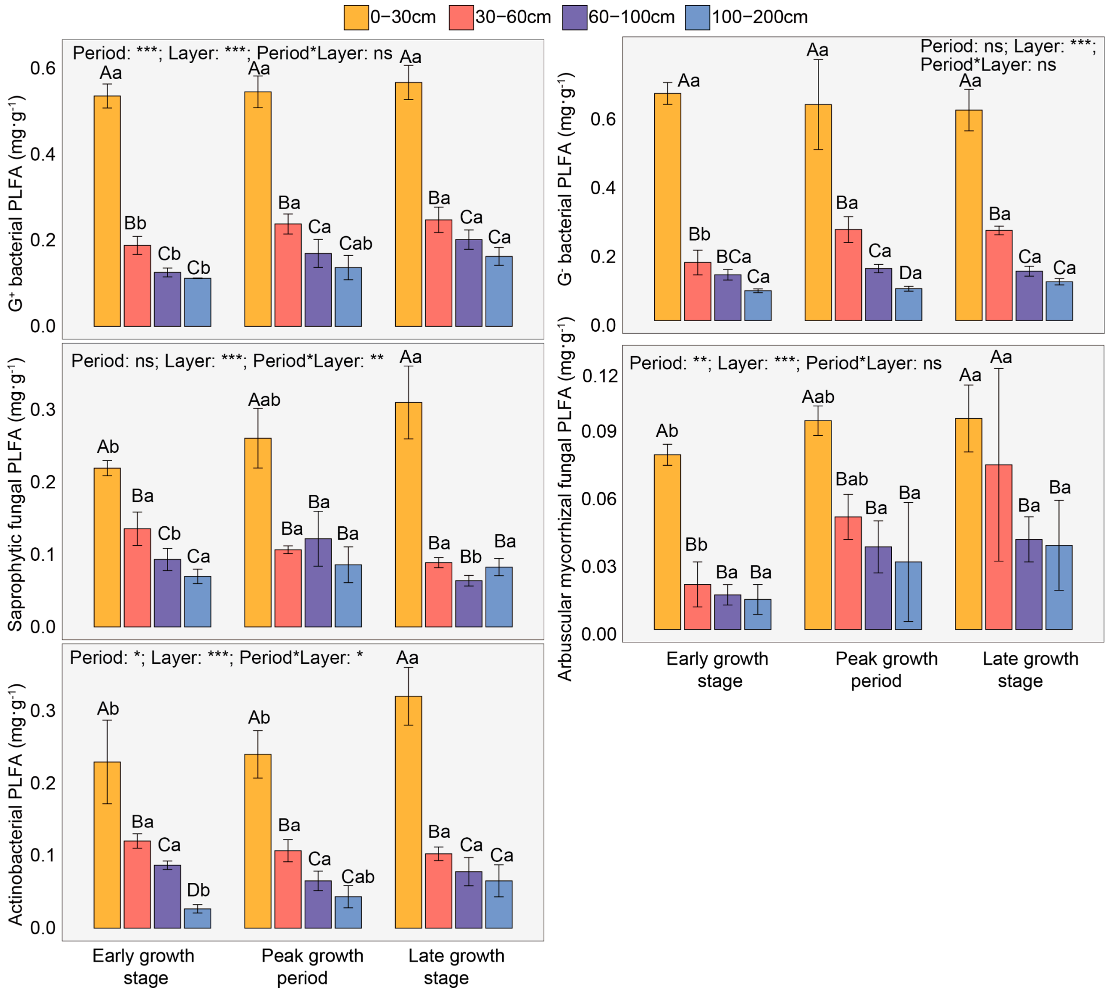

Figure S2. Variations in soil microbial phospholipid fatty acid (PLFA) across different periods and soil layers. Uppercase letters indicate significant differences among soil layers within the same period, while lowercase letters denote significant differences across periods within the same soil layer ($p < 0.05$). Based on two-way ANOVA, asterisks represent the following significance levels: * $p < 0.05$, ** $p < 0.01$, *** $p < 0.001$.

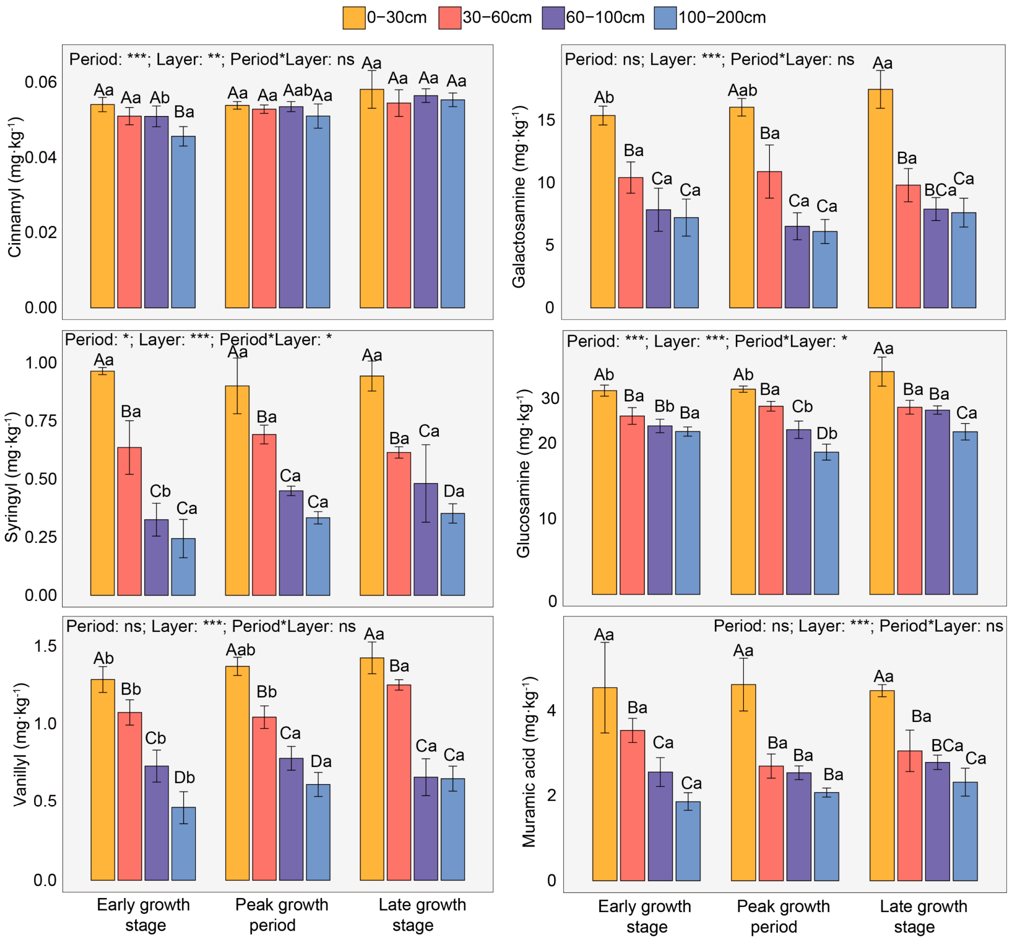

Figure S3. Variations in soil amino sugars and lignin phenols across different periods and soil layers. Uppercase letters indicate significant differences among soil layers within the same period, while lowercase letters denote significant differences across periods within the same soil layer ($p < 0.05$). Based on two-way ANOVA, asterisks represent the following significance levels: * $p < 0.05$, ** $p < 0.01$, *** $p < 0.001$.

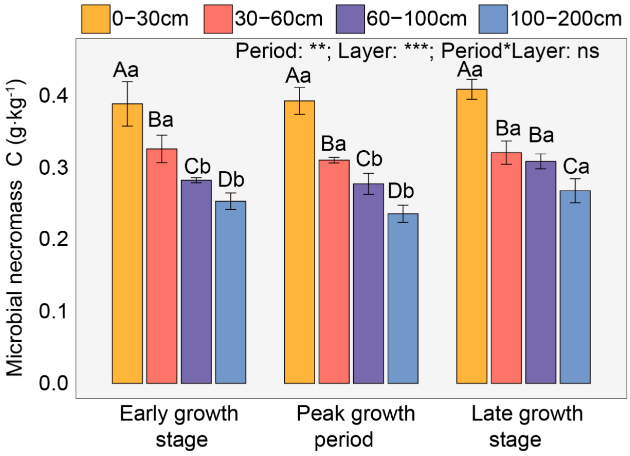

Figure S4. Variations in soil microbial necromass carbon across different periods and soil layers. Uppercase letters indicate significant differences among soil layers within the same period, while lowercase letters denote significant differences across periods within the same soil layer ($p < 0.05$). Based on two-way ANOVA, asterisks represent the following significance levels: * $p < 0.05$, ** $p < 0.01$, *** $p < 0.001$.

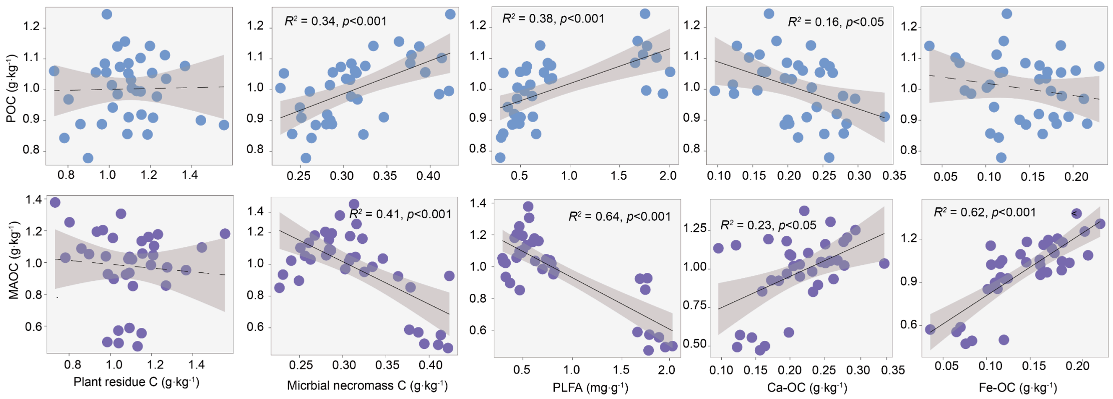

Figure S5. Relationships between particulate organic carbon (POC), mineral-associated organic carbon (MAOC) and plant residue carbon, microbial necromass carbon, phospholipid fatty acids (PLFA), calcium-bound organic carbon (Ca-OC), iron-bound organic carbon (Fe-OC). The shaded area represents the 95% confidence interval, and the dashed line indicates the interval where $p > 0.05$.

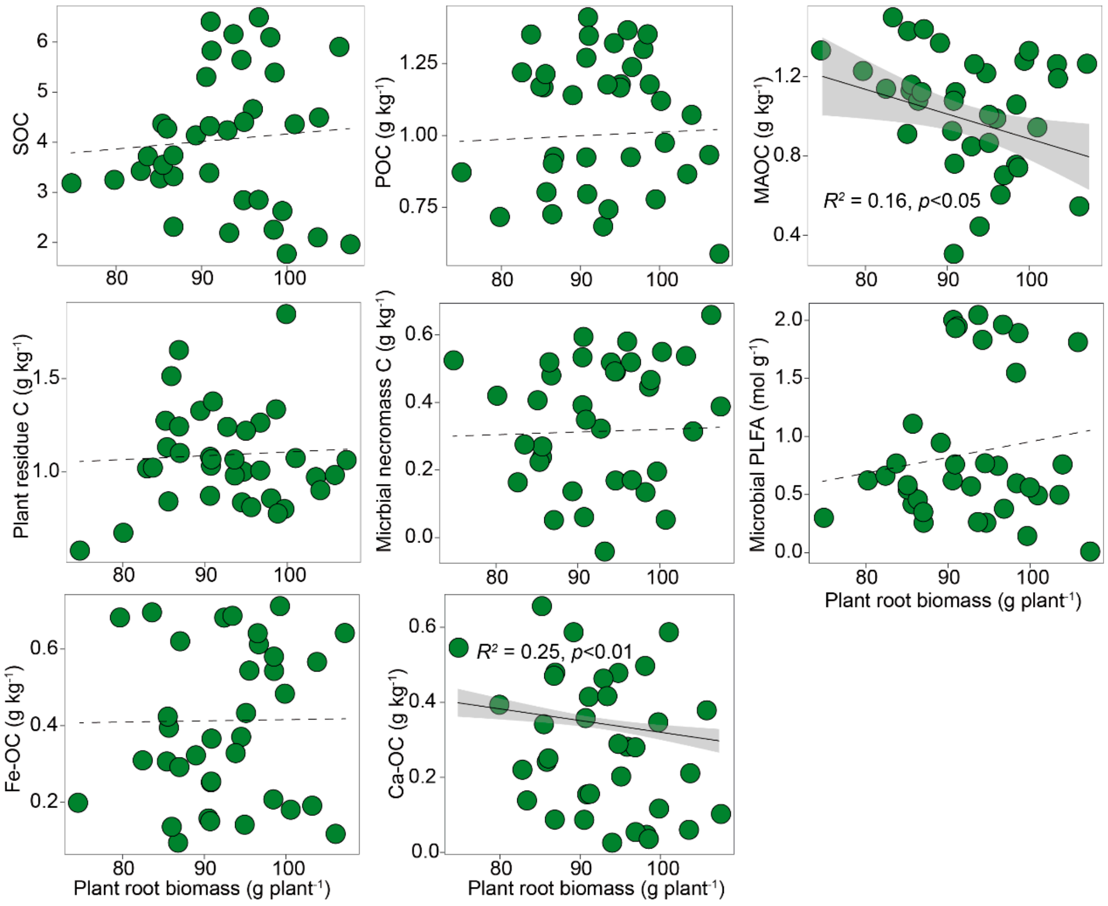

Figure S6. Relationships between plant root biomass and particulate organic carbon (POC), mineral-associated organic carbon (MAOC) and plant residue carbon, microbial necromass carbon, phospholipid fatty acids (PLFA), calcium-bound organic carbon (Ca-OC), iron-bound organic carbon (Fe-OC). The shaded area represents the 95% confidence interval, and the dashed line indicates the interval where $p > 0.05$.
